# Rebound Catatonia Associated With Injectable Paliperidone

**DOI:** 10.7759/cureus.40478

**Published:** 2023-06-15

**Authors:** Bamidele O Johnson, Godwin Orji, Olayemi O Johnson, Jacky Petion, Oluwaseun Oke, Sana Elham Kazi, Christian Nwabueze, Ayodeji Jolayemi

**Affiliations:** 1 Psychiatry, Interfaith Medical Center, Brooklyn, USA; 2 Psychiatry, Astute Healthcare Services, LLC, Bowie, USA

**Keywords:** bush-francis catatonia rating scale, ect, cyp system, cyp, long acting injectable, neuroleptic malignant syndrome (nms), stupor, excited catatonia, malignant catatonia, catatonia

## Abstract

Paliperidone is an atypical antipsychotic medication commonly used to treat schizophrenia, schizoaffective disorder, and bipolar disorder. It is a metabolite of risperidone and has a similar mechanism of action, primarily blocking dopamine 2 receptors (D2 receptors) in the brain. Paliperidone has various adverse effects, including extrapyramidal symptoms, weight gain, and metabolic disturbances. Catatonia is rare but severe side effects can occur in the context of an underlying psychiatric, neurologic, or general medical condition. Paradoxically, antipsychotics for treating schizophrenia or bipolar spectrum disorders can precipitate or worsen catatonic symptoms. The report suggests that 17-19% of all cases diagnosed as catatonia due to other medical conditions are medication-induced. Catatonia is a neuropsychiatric syndrome that presents as a cluster of psychomotor signs and symptoms resulting in movement and behavior aberrations. Various symptoms, including mutism, stupor, rigidity, and abnormal movements, characterize catatonia. Catatonia is a potentially life-threatening condition requiring prompt recognition and management. Here, we present a case of a patient with catatonia associated with long-acting injectable paliperidone intramuscular therapy in a patient with schizophrenia.

## Introduction

Paliperidone is a commonly prescribed second-generation (atypical) antipsychotic medication used for various mental health conditions, such as schizophrenia, schizoaffective disorder, bipolar disorder, and certain types of depression [1]. It is derived from risperidone, approved by the US Food and Drug Administration (FDA) in 1993, and is widely prescribed worldwide [2]. Paliperidone, also known as 9-hydroxy-risperidone, was approved by the FDA in 2006 and is a more recent addition to the medication options [1,3]. Despite minor differences in their pharmacodynamic properties, risperidone and paliperidone share the same receptor-blocking effect profile, predominantly on dopamine (D2) in the mesolimbic pathway and serotonin (5HT2A) receptors in the prefrontal cortex of the brain [4]. These receptor pathways between the basal ganglia, limbic systems, and frontal cortex are affected in schizophrenia and related disorders [4]. Paliperidone, however, has a higher affinity for dopamine receptors, making it potentially more effective in treating positive symptoms of schizophrenia like hallucinations and delusions [5,6].

Regarding pharmacokinetics, paliperidone has a longer half-life and is eliminated from the body at a slower rate than risperidone [7,8]. This means paliperidone can be dosed less frequently, improving medication compliance [3,7,8]. Paliperidone is available in extended-release injection and tablet forms, while risperidone is available in tablet, oral solution, and extended-release injection forms [9]. Paliperidone has a wide distribution throughout the body and reaches peak plasma concentration approximately 24 hours after dosing [7,8]. It binds to various receptors in the body, including alpha-1 adrenergic, H-1 histaminergic, and alpha-2 adrenergic receptors, contributing to its overall effects [5]. Paliperidone's metabolism in the liver is not as extensive as risperidone. It may be advantageous for individuals with liver problems as it is less likely to cause significant interactions with other medications [3,7,10]. Both risperidone and paliperidone are primarily excreted through the kidneys [11].

Paliperidone palmitate, an ester prodrug of paliperidone, is a long-acting injectable formulation used to treat schizophrenia [8]. It is available in different durations of action, including one-month, three-month, and six-month injectable forms [8,12]. The palmitate ester makes the medication less soluble, resulting in extended-release properties and allowing for less frequent dosing [13]. Paliperidone palmitate is indicated for individuals who have already shown tolerance to oral paliperidone or risperidone and can be used as monotherapy or in conjunction with mood stabilizers [8]. Dosage may vary based on individual factors, such as kidney function and the clinician's assessment [8,13].

Common side effects of paliperidone include drowsiness, dizziness, constipation, nausea, weight gain, increased appetite, dry mouth, headache, anxiety, and insomnia [9,11]. It can also cause extrapyramidal symptoms, hyperprolactinemia, sexual dysfunction, pancreatitis, restlessness, blood pressure or heart rate changes, and in rare cases, neuroleptic malignant syndrome [1,3].

Catatonia is a rare but severe neuropsychiatric syndrome characterized by a cluster of psychomotor signs and symptoms that result in abnormal movement and behavior [14]. It presents various manifestations, including rigidity, immobility, mutism, stupor, abnormal movement, and bizarre posturing [15]. The current diagnostic criteria in the Diagnostic and Statistical Manual of Mental Disorders, 5th Edition (DSM-5) require the presence of three or more symptoms, such as stupor, catalepsy, mutism, posturing, negativism, stereotypes, mannerisms, grimacing, agitation, echopraxia, and echolalia [15,16].

More than 40 clinical signs are associated with catatonia, and several scales have been proposed for its assessment. The preferred rating scale for routine evaluation is the 23-item Bush-Francis Catatonia Rating Scale (BFCRS) due to its proven validity, reliability, and ease of use. Catatonia is identified using two or more items from the first 14 items of the BFCRS [16].

Catatonia can be classified as malignant or non-malignant catatonia. Malignant catatonia is a life-threatening form that involves fever, rigidity, and autonomic instability [17]. Catatonia can manifest as either retarded catatonia, characterized by stupor and withdrawal symptoms, or excited catatonia, characterized by excessive and purposeless motor activity [17,18].

The etiology of catatonia is multifactorial, including medication side effects, and it can be life-threatening in some cases [18]. Catatonia can occur in the context of underlying psychiatric, neurological, or general medical conditions [14,15,17]. The incidence of catatonia in acute inpatient psychiatric settings has been reported to be between 5% and 20% [14]. Catatonia has been associated with schizophrenia, related disorders, and major depressive disorder. Symptoms of catatonia can fluctuate during episodes, with periods of withdrawal and excitation [14,16,17].

Reports suggest that 17-19% of catatonia cases attributed to medical etiology are medication-induced [18]. Therefore, recognizing catatonia is crucial as it can be caused or worsened by treating the underlying disorder. For example, antipsychotic drugs to treat bipolar or psychotic disorders can exacerbate catatonia [14]. Risk factors for developing antipsychotic-induced catatonia include a prior history of catatonia, high doses of antipsychotics, and multiple antipsychotic medications [17,18]. Paradoxically, antipsychotics to treat schizophrenia or bipolar spectrum disorders can precipitate or worsen catatonic symptoms [14,17]. Certain medical conditions, such as central nervous system infections or autoimmune diseases, may also increase the risk of developing catatonia [18].

Catatonia can lead to medical complications associated with high morbidity and mortality [19]. Despite improved recognition and treatment, malignant catatonia has a mortality rate of approximately 9-10% [18]. Medical complications associated with catatonia include dehydration, acute renal failure, urinary retention, aspiration pneumonia, deep venous thrombosis, pulmonary embolism, rhabdomyolysis, hypoglycemia, cachexia, EKG changes, respiratory arrest, myocardial infarction, cardiac arrest, and death [19,20].

## Case presentation

The patient was a 27-year-old African-American male, single, unemployed, supported by family, domiciled with his mother in an apartment, unemployed with a past psychiatric history of schizophrenia and a past medical history of prediabetes and obstructive sleep apnea. The patient was brought into the emergency department by EMS, which his mother activated for bizarre behavior in the context of medication non-compliance.

On assessment in the emergency department (ED), the patient appeared catatonic. He was mute, staring at the clinician, and exhibited blunted facies. The patient had a score of 17 on BFCRS. Positive items on the BFCRS included immobility/stupor (+1), mutism (+2), staring (+2), posturing/cataplexy (+2), grimacing (+1), stereotypy (+1), withdrawal (+1), automatic disobedience (+1), ambitendency (+3), and grasp reflex (+3). The patient received midazolam 2 mg intramuscularly thrice daily for catatonia. The patient responded to the benzodiazepine challenge test and was more responsive and communicative afterward.

In the ED, he had a temperature of 36.3°C (97.9°F), pulse of 106 beats per minute, blood pressure of 120/81 mm Hg, respiratory rate of 16 breaths per minute, and oxygen saturation of 96% on room air. His weight was 255 lb (115.7 kg), with a BMI (calculated) of 35.6. There was no associated autonomic instability noted.

A physical exam showed an alert, disheveled-looking male who appeared older than the stated age and not in acute distress. His pupils were equal, round, and reactive to light; he would not comply with extraocular muscle testing. He did open his mouth on request and stuck out his tongue midline; there was no erythema, and mucous membranes were moist. The cardiopulmonary exam was unremarkable; abdominal palpation did not cause any change in his facial expression, and he was soft without masses. While he complied with several requests for the cranial nerve exam, he would not move his fingers or toes when asked but was noted to turn his head in all directions and roll from side to side, moving all extremities equally. His brachioradialis and Achilles reflexes were equal, and Babinski's reflexes were downgoing. He exhibited stupor and mutism with fixed postures.

On mental status examination, the patient was a young African American male who appeared older than the stated age. The patient appeared unkempt, disheveled-looking, and with poor grooming. Significant psychomotor retardation was noted. The speech was mute. The patient exhibited blunted facies, blank stares, and poor eye contact. His affect was blunt and flat. Due to his limited interactions, the clinician could not assess other domains involving his thought process, thought content, perceptual disturbances, mini-mental status examination, insight, and judgment.

Finger-stick glucose was 127 mg/dL. Glycosylated hemoglobin (HbA1c) was 6.0%. Non-contrast head computed tomography (CT) was performed due to concern for stroke, which showed calcifications along the falx and was otherwise unremarkable. Laboratory tests done are shown in Table 1.

**Table 1:** Results of laboratory tests on admission

Investigations	Results	Normal range
Blood urea nitrogen (BUN)	12.8	6.0-23.0 mg/dL
Creatinine (Cr)	0.78	0.50-1.20 mg/dL
Sodium (Na)	139	136-145 mmol/L
Potassium (K)	4.7	3.5-5.0 mmol/L
Calcium (Ca)	10.4	8.5-10.5 mg/dL
Chloride (Cl)	106	96-106 mmol/L
Bicarbonate (HCO3)	27.1	20-28 mmol/L
Alanine transaminase (ALT)	67	0-33 U/L
Aspartate transaminase (AST)	30	5-32 U/L
Alkaline phosphatase (ALP)	78.8	35-104 U/L
White blood count (WBC)	11.4	4.5-11.0 10x3/UL
Lymphocytes	14.4%	22-48%
Monocytes	8.3%	2-14.0%
Eosinophils	0.1%	0.5-5.0%
Neutrophils	76.8	40-70%
Absolute neutrophil % (ANC)	8.7%	2.00-7.9 10x3/UL
Hemoglobin (Hgb)	15.2	12.0-16.0
Hematocrit (HCT)	47.5	39-53%
Platelet (PLT)	322	130-400 10x3/UL
Blood alcohol level (BAL)	0.0	0.0-14.0 mg/dL
Urine toxicology (Urine tox)	Negative	
Urine specific gravity	>1.030	1.005-1.030
Thyroid-stimulating hormone (TSH)	2.650	0.465-4.680 UIU/mL
Free thyroxine (FT4)	1.11	0.61-1.12 ng/dL
Prolactin	40	4-15.2 ng/mL
Cholesterol	202	120-200 mg/dL
Triglycerides (TG)	153	<150 mg/dL
High-density lipoprotein (HDL)	44	60-80 mg/dL
Low-density lipoprotein (LDL)	127.6	50-130 mg/dL
Serum creatinine kinase (CK)	1,858.9	30-200 U/L
Hemoglobin A1c (A1c)	6.0	4.8-5.6%

Urinalysis (UA)	Trace ketones, UA protein 100 mg/dL, negative nitrites, negative leukocyte esterase, negative glucose, and negative ketones

The following day, the patient was seen and evaluated in the inpatient unit. The patient's catatonic symptoms improved on transfer to the inpatient unit as he began to talk a little but exhibited psychomotor retardation. The patient seemed to be a poor historian. The patient reported "I have schizophrenia," and stated he was in his usual state of health until one week ago when he started hearing voices. He reported the voices were many. He says, "The voices were both talking among themselves and discussing me." He denied any commanding instructions from the voices. He denied visual hallucinations. He reported that shampoo entered his eyes three days before his hospitalization, he had his contact on, and his eyes became blurry afterward. He said that the situation worsened his auditory hallucinations. The patient endorsed thought insertion, thought withdrawal, and ideas of reference. He endorsed paranoid delusion stating, "I feel people are after me and want to kill me." He reported not taking his medication since he ran out two weeks ago. He said he had received the Invega shot monthly for about three years, and he goes to the ED for the shots. He reported his last shot was a month ago. He denied suicidal ideation, intent, or plan. He denied homicidal ideations, intents, or plans.

The patient reported eight previous hospitalizations for schizophrenia since 2018 when he received the diagnosis at age 23. His most recent hospitalization was about eight months before the current presentation. Past medication trials include oral doses of 5 mg of haloperidol twice daily, Depakote 500 mg twice daily, and Cogentin 1 mg twice daily for extrapyramidal symptom prevention. The patient reports his home medications were risperidone 3 mg oral dose twice daily, Invega Sustenna 156 mg intramuscular long-acting monthly injectable, and melatonin 5 mg oral dose at bedtime for insomnia. He has not been compliant with his medication since running out on his medications two weeks ago. Serum creatinine kinase level was trending upwards (2160 U/L).

Due to improved catatonia, intramuscular midazolam 2 mg was discontinued, and Ativan 2 mg oral dose three times daily and risperidone 1 mg oral dose twice daily were started for psychosis on the third day.

The patient continued to exhibit psychomotor retardation. He appeared to have no muscle stiffness and showed good muscle power in his upper and lower limbs. On the fifth day, the oral frequency dose of Ativan was tapered from 2 mg thrice daily to 2 mg twice daily due to improvement in his catatonic symptoms.

Due to his psychotic symptoms, including auditory hallucinations, paranoid delusions, and disorganized behavior, his oral dose of risperidone was gradually up-titrated to 3 mg twice daily by the 11th day.

By the 11th day, his auditory hallucination and paranoid delusion had improved significantly. He began to take showers and attend select group therapy sessions. He continued to adhere to medications. He agreed to take the long-acting injectable after psycho-educating about the benefits and side effects of the long-acting injectable. He received Invega Sustenna (paliperidone palmitate) 234 mg intramuscularly and 156 mg intramuscularly on the 11th and 15th day, respectively.

On the 15th day, the patient had a rebound psychosis. He became catatonic again, exhibiting mutism, fixed posturing, staring into space, immobility, and inability to get out of bed. Vital signs were normal with no signs of autonomic instability. The patient scored 16 on BFCRS and began to hear voices again. Positive items on BFRCS included immobility/stupor (+2), mutism (+3), staring (+2), posturing/cataplexy (+2), grimacing (+3), withdrawal (+1), and ambitendency (+3). His behavior became disorganized, and he endorsed auditory hallucinations of antagonizing voices. He was restarted on an oral dose of Ativan 2 mg twice daily for catatonia. The patient's catatonic symptoms gradually improved within the next hour as he became more conversational and mobile on the unit.

On the 19th day, his oral dose of risperidone 3 mg twice daily was changed to an oral dose of aripiprazole 10 mg daily due to the possibility of rebound psychosis, including catatonic symptoms, auditory hallucinations, and paranoid delusions. Oral melatonin 6 mg was prescribed for insomnia at bedtime. The patient was explained each treatment's risks, benefits, and alternatives. The patient was able to communicate his understanding and agreed to take the medications.

On the 25th day, his mood and affect became brighter, and he became less disorganized. His catatonic symptoms were improving. He denied auditory hallucinations. The patient was seen attending group activities again. He agreed to continue with monthly long-acting intramuscular (aripiprazole lauroxil) Abilify Maintena 400 mg injection for psychosis instead of the Invega Sustenna shot due to the possibility of rebound psychosis. His next shot of Abilify Maintena (aripiprazole lauroxil) 400 mg intramuscular dose was scheduled for the next month. During the eight months following his discharge, his psychotic symptoms were further controlled with an oral dose of aripiprazole 20 mg daily without relapse or worsening.

## Discussion

Catatonia is a clinical phenomenon observed in patients with various psychiatric disorders, such as schizophrenia, schizoaffective disorder, and bipolar disorder [14,15]. Studies indicate that approximately 30% of individuals diagnosed with schizophrenia and 45% of those with bipolar disorder experience catatonia [18]. Additionally, catatonia can be associated with other psychiatric conditions, including depression, obsessive-compulsive disorder, post-traumatic stress disorder, autism spectrum disorder, and withdrawal from alcohol or benzodiazepines [14,17].

In our case, the patient exhibited catatonic features and had a psychiatric history of schizophrenia. The patient's mother and chart reviews indicated no prior episodes of catatonia. However, given the patient's diagnosis of schizophrenia, it is likely that catatonia was associated with this underlying psychiatric illness, as catatonia is observed in approximately 30% of patients with schizophrenia [14,18].

It is important to note that catatonia can also occur in the context of general medical or neurological conditions [18]. About 25% of catatonic cases are related to medical or neurological causes [14,17,18]. Therefore, conducting a comprehensive evaluation to exclude underlying medical causes of catatonia is essential.

In this case, the patient underwent head CT scans and further laboratory investigations to rule out other medical causes of catatonia. The results did not reveal any alternative medical etiology. However, the patient presented with an elevated serum creatinine phosphokinase, a non-specific marker that can result from muscle injuries accompanying immobility due to the catatonic state. The absence of fever and autonomic instability ruled out neuroleptic malignant syndrome (NMS), considered an iatrogenic subtype of malignant catatonia by some researchers [17,19]. Although NMS remains a differential diagnosis in patients with catatonia due to shared clinical features, it requires distinct treatment strategies [17-19].

A comprehensive workup for catatonia may involve various tests, including complete blood count, comprehensive metabolic panel, thyroid panel, urinalysis, urine toxicology, ESR, serum creatinine phosphokinase, serum iron, antinuclear antibody (ANA) panel, CT, MRI, EEG, lumbar puncture, CSF analysis, and other clinically relevant studies. It is crucial to consider a broad differential and conduct a thorough assessment when evaluating patients presenting with catatonia in the emergency department [18,20].

Promptly recognizing treatable causes of catatonia is crucial, as addressing the underlying causes often leads to the resolution of symptoms [14,17]. It is important not to rely solely on the patient's psychiatric history when diagnosing catatonia but to consider other potential causes. Research suggests that approximately 17-19% of catatonia cases can be attributed to other medical conditions, including medication-induced catatonia [18].

In this case, the possibility of medication-induced catatonia was considered, primarily due to using long-acting injectable paliperidone, an atypical antipsychotic. Previous studies have shown that typical or first-generation antipsychotics like haloperidol and fluphenazine are associated with catatonia [15,16]. The role of atypical antipsychotics in catatonia treatment remains controversial [16,18]. Some studies suggest potential benefits due to their weak GABAergic action and 5-HT2 serotonergic antagonism, leading to dopamine release in the prefrontal cortex and improved catatonia [14,17]. However, conflicting evidence suggests that atypical antipsychotics may exacerbate catatonia [18].

Both typical and atypical antipsychotics can contribute to maintaining or worsening catatonia and increase the risk of developing NMS, with a higher risk associated with neuroleptics that have strong D2 antagonism [17,19]. Therefore, in cases of neuroleptic-induced catatonia, it is generally recommended to discontinue the antipsychotic and administer benzodiazepines or electroconvulsive therapy (ECT) [14,19,20]. Other treatments include zolpidem, a positive allosteric modulator of the GABA-A receptor, glutamate antagonists, anti-epileptic drugs such as valproic acid and carbamazepine, and repetitive transcranial magnetic stimulation (rTMS) [18,20].

In this case, the patient responded favorably to a trial of intramuscular benzodiazepine. However, due to ongoing psychosis, risperidone, an atypical antipsychotic, was gradually reintroduced while benzodiazepines were continued. Nonetheless, the patient experienced rebound catatonia and psychosis following a long-acting intramuscular paliperidone injection, further supporting the suspicion of paliperidone-induced catatonia.

However, it is also essential to consider the possibility of overprescription contributing to the patient's catatonic symptoms. The patient had been stabilized on a monthly dose of Invega Sustenna (paliperidone palmitate) at 156 mg. According to the prescribing information for Invega Sustenna, the recommended dose is the patient's previously stabilized dose in cases with a gap of fewer than six weeks since the last dose [21]. In this case, the patient's stabilized dose was 156 mg. However, the patient initially received a higher dose of 234 mg, followed by the previously stabilized dose of 156 mg after only four days, despite the gap between doses being less than six weeks.

Deviation from the recommended dosing schedule and an increase in dosage could potentially contribute to the emergence of catatonic symptoms. Adhering to the recommended dosing guidelines for medications is crucial to ensure optimal therapeutic outcomes and minimize the risk of adverse effects [21]. Healthcare providers must follow the recommended dosing guidelines to ensure patient safety and maximize treatment effectiveness.

Atypical antipsychotics may have a role in cases with residual psychotic symptoms such as delusions and hallucinations, especially in schizophrenia, or as a prophylactic treatment in psychosis and mood disorders [16,18,20]. Atypical antipsychotics with low D2 blockade, such as quetiapine, olanzapine, or aripiprazole with D2 partial agonism, should be preferred in these populations [18,20].

Consequently, benzodiazepine therapy was reinstated, and the patient responded well to this treatment. Following the resolution of catatonic symptoms, the patient was initiated on oral aripiprazole, and later, he received monthly long-acting intramuscular aripiprazole injections without any reported catatonia during eight months of follow-up in the outpatient clinic.

In addition to pharmacotherapy, supportive care measures were implemented to manage the patient's catatonic symptoms. Adequate hydration, nutrition, and a safe environment were ensured, and regular turning and repositioning were conducted to prevent complications associated with immobility.

A multidisciplinary approach was adopted throughout the patient's hospitalization, involving psychiatric follow-up consultations, close physical health monitoring, and regular laboratory tests to assess metabolic profile and potential complications related to catatonia.

## Conclusions

This case study underscores the intricate connection between catatonia and underlying psychiatric disorders, particularly schizophrenia, while drawing attention to the significance of considering medication-induced catatonia, such as the potential link to long-acting injectable paliperidone. Overprescription of paliperidone palmitate may also contribute to developing or exacerbating catatonic symptoms. Adherence to recommended dosing guidelines and a multidisciplinary approach are crucial for accurate diagnosis and optimal management of catatonia.

The diagnosis and management of catatonia require a comprehensive approach that takes into account various potential causes, promptly identifies treatable factors, and employs a combination of pharmacotherapy and supportive care. Healthcare providers should remain vigilant in assessing for catatonic symptoms, particularly in patients with a history of catatonia or those at a high risk of developing it. Timely intervention, which may include the use of benzodiazepines or ECT alongside supportive measures, can lead to positive outcomes and improve the overall well-being of the patient. To optimize outcomes and prevent relapse, collaborative efforts among healthcare professionals, active involvement of patients and their families, and a well-structured aftercare plan are essential.
